# The Committee on Legislation

**Published:** 1883-07

**Authors:** 


### The Committee on Legislation.

This committee has begun its work with method and determination. Correspondence was at once instituted with the authorities of every State in the Union having a medical practice act, and with the proper officer of all established Boards, asking for information upon the practical workings of their respective bodies. The information thus gathered, the committee hope to embody in a bill, and is confident that when such a bill is prepared, it will be worthy of the support of the profession. We trust that in our next issue we can give our readers at least a synopsis of what we firmly believe will be the most important medical law of this State.

The fact that State Examining Boards are now in operation in Illinois, West Virginia, North Carolina, Alabama, Delaware, Minnesota and Missouri, in a great measure simplifies the labor of preparing such a bill for our own State, and also furnishes an incentive for vigorous action in this direction. The profession of the Empire State should not be the last to take this important step, and we are glad that the physicians of Erie county have inaugurated the movement.

